# Physiological responses of mechanosensory systems in the head of larval zebrafish (*Danio rerio*)

**DOI:** 10.3389/frobt.2023.1212626

**Published:** 2023-07-31

**Authors:** Nils Brehm, Nils Wenke, Keshia Glessner, Melanie Haehnel-Taguchi

**Affiliations:** Department of Developmental Biology, Institute for Biology 1, University of Freiburg, Freiburg, Germany

**Keywords:** lateral line, mechanosensation, zebrafish, trigeminal, statoacoustic

## Abstract

The lateral line system of zebrafish consists of the anterior lateral line, with neuromasts distributed on the head, and the posterior lateral line, with neuromasts distributed on the trunk. The sensory afferent neurons are contained in the anterior and posterior lateral line ganglia, respectively. So far, the vast majority of physiological and developmental studies have focused on the posterior lateral line. However, studies that focus on the anterior lateral line, especially on its physiology, are very rare. The anterior lateral line involves different neuromast patterning processes, specific distribution of synapses, and a unique role in behavior. Here, we report our observations regarding the development of the lateral line and analyze the physiological responses of the anterior lateral line to mechanical and water jet stimuli. Sensing in the fish head may be crucial to avoid obstacles, catch prey, and orient in water current, especially in the absence of visual cues. Alongside the lateral line, the trigeminal system, with its fine nerve endings innervating the skin, could contribute to perceiving mechanosensory stimulation. Therefore, we compare the physiological responses of the lateral line afferent neurons to responses of trigeminal neurons and responsiveness of auditory neurons. We show that anterior lateral line neurons are tuned to the velocity of mechanosensory ramp stimulation, while trigeminal neurons either only respond to mechanical step stimuli or fast ramp and step stimuli. Auditory neurons did not respond to mechanical or water jet stimuli. These results may prove to be essential in designing underwater robots and artificial lateral lines, with respect to the spectra of stimuli that the different mechanosensory systems in the larval head are tuned to, and underline the importance and functionality of the anterior lateral line system in the larval fish head.

## 1 Introduction

To survive and prosper in aquatic environments, it is essential for animals to have a sensory system that is tuned to different types of water motion, like laminar or turbulent flow fields. Especially in murky water or during the night, when visual information is lacking, animals need to rely on information gained from hydrodynamic stimuli rather than vision alone. Aquatic animals like fish and amphibians have evolved a system called the lateral line that enables flow field perception (Dijkgraaf, 1963) and facilitates a wide variety of behaviors. For example, the lateral line system plays an important role in schooling (Pitcher et al., 1976), prey capture (Coombs and Conley, 1997; Montgomery et al., 2002), predator avoidance (Stewart and McHenry, 2010; Stewart et al., 2013), and rheotaxis (Oteiza et al., 2017). Most of these behaviors occur already in larval zebrafish. Flow fields contain important information about the physical properties of the animal’s surroundings. This includes the shape and structure of the underwater habitat and other animals moving through the water, like conspecifics, prey, or predators. Through the lateral line system, fish can detect these hydrodynamic cues and use them to make behavioral decisions (Bleckmann and Zelick, 2009). For example, the detection of hydrodynamic pressure gradients that are generated by nearby prey movements allows predators to localize and approach the prey, thus improving their hunting success (Coombs and Conley, 1997; Montgomery et al., 2002; Carrillo and McHenry, 2016; Carrillo et al., 2019). Similarly, a prey itself may rely on detecting the wakes produced by approaching or striking predators (Stewart and McHenry, 2010; Stewart et al., 2013). In fish species that show social behaviors like schooling, the lateral line is crucial for synchronizing all movements of the individuals and, thus, the entire school by detecting and predicting the water motion produced by neighboring conspecifics (Pitcher et al., 1976; Faucher et al., 2010).

**FIGURE 1:**
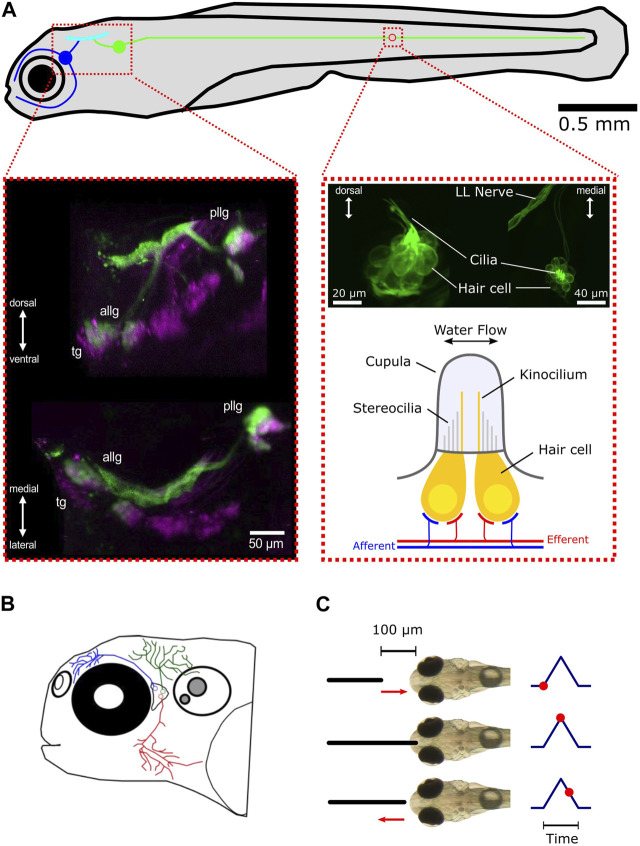
Overview of the lateral line and trigeminal system in zebrafish larvae. **(A)** Schematic representation of the lateral line system and its subsystems, the anterior lateral line (blue), and the posterior lateral line (green). The blue and green circles represent the anterior lateral line and posterior lateral line ganglia, respectively. All afferent neurons are located in the ganglia and receive their sensory inputs from neuromasts while sending signals via ascending projections into the hindbrain (cyan). For the sake of simplicity, only one neuromast (red circle) is shown. Left inset: maximum intensity z-projection of a larva expressing mCherry (magenta) driven by islet2B and SILL:GFP (green). Anterior lateral line ganglion (ALLG), posterior lateral line ganglion (PLLG), and trigeminal ganglion (TG). Shown are a transverse plane (bottom) and a sagittal plane (top). Right inset (top): maximum intensity z-projection of a larva expressing GFP (Brn3c:GFP) in hair cells. The left side shows a lateral view of a neuromast. The right side shows a dorsal view. Right inset (bottom): schematic representation of a neuromast. For convenience, only two hair cells are depicted (in yellow). Afferent connections from the ganglia neurons are shown in blue. Innervation by different efferent systems is illustrated in red. **(B)** Schematic overview of the trigeminal system in the zebrafish larvae. Shown are three representative trigeminal afferent neurons with their arborizations and fine nerve endings covering the skin of the head. **(C)** Schematic demonstration of the tactile stimulation procedure. The black solid line depicts the platinum wire used for touching the front head. The default starting position of the wire is 100 µm away from the skin. It then approaches the front head with a preset speed, touches, and slightly impresses the skin for a preset of time and then returns back to the starting position. The corresponding time points are shown on the right as red dots.

The lateral line in fish consists of two main structures: the sensory receptor units called neuromasts and the afferent neurons contained in a ganglion that process and forward the information received by the neuromasts to higher brain areas (Figure 1A). The hair cells contained in neuromasts are sensitive to the deflection of the cupula. Each hair cell has structures that increase the membrane surface, like stereocilia and kinocilia that protrude into the gelatinous cupula (Bleckmann and Zelick, 2009; Nicolson, 2017). When the long kinocilium is deflected against the stereocilia, ion channels open, and the resulting influx of ions changes the membrane potential. Such a deflection leads to the depolarization of the hair cell, which then triggers the release of the neurotransmitter glutamate onto the primary sensory afferent neurons (Hudspeth and Corey, 1977; Trapani and Nicolson, 2011). Each hair cell has a deflection direction that it is most sensitive to, and every neuromast consists of two opposing polarity subgroups of hair cells (Nagiel et al., 2008; Faucherre et al., 2009; Lozano-Ortega et al., 2018), making the neuromasts a bi-directional sensory unit. An individual lateral line afferent neuron only innervates hair cells of the same polarity and can receive inputs from multiple neuromasts. On the other hand, every neuromast may be innervated by up to four different afferent neurons (Faucherre et al., 2009). In fish, there are two different types of neuromasts: superficial and canal neuromasts. Superficial neuromasts lie on the surface of the skin and have direct contact with the surrounding water volume (Mogdans and Bleckmann, 2012; Mogdans, 2019). Local three-dimensional water motion with a certain speed and directionality (local flow field) around the neuromast will directly act on the neuromast’s cupula and deflect it, depending on the mechanical properties of the cupula. Therefore, superficial neuromasts can be seen as velocity detectors (Haehnel-Taguchi et al., 2014; Pichler and Lagnado, 2019; De Faveri et al., 2021). The second type of neuromast is not located on the surface of the skin but recedes under the skin during development into canals, connected to the surrounding water by pores (Bleckmann and Zelick, 2009). Being shielded from the local water flow in this way makes them sensitive to pressure differences between the canal and the outside water volume. As such, canal neuromasts are considered to be pressure rather than flow sensors. However, in this study, we will use zebrafish possessing only superficial neuromasts during their larval stage. Therefore, we will concentrate on superficial neuromasts in the following. In contrast to the adult stage, zebrafish larvae only have a small number of neuromasts distributed over the entire body. The combination of available genetic tools and translucence of the body makes the zebrafish larva the ideal model organism to study water flow perception in animals. The lateral line system is further anatomically subdivided into the anterior lateral line (ALL) and the posterior lateral line (PLL) [Figure 1A (top: blue and green)]. The posterior lateral line hair cells are arranged in sequence along the trunk midline (Metcalfe et al., 1985), and most of them have their highest directional sensitivity parallel to the rostro-caudal body axis (Pujol-Martí and López-Schier, 2013). In contrast to the posterior lateral line, the anterior system is composed of cranial neuromasts with a more complex anatomical arrangement (Raible and Kruse, 2000) and directional tuning (Chou et al., 2017). The primary afferent neurons of the lateral line are located in the posterior lateral line ganglion (PLLG) and the anterior lateral line ganglion (ALLG). From there, they project into the medial octavolateralis nucleus (MON) in the hindbrain (Metcalfe et al., 1985).

Alongside the lateral line, the trigeminal system, with its fine nerve endings innervating the skin, could also contribute to perceiving mechanosensory stimulation and thus play an additional role in perceiving water flow. Together with Rohon–Beard neurons, they are the earliest mechanosensory cells to develop in the zebrafish larva and are known to be involved in escape behavior (Metcalfe et al., 1990; Douglass et al., 2008). The trigeminal afferent neurons are contained in the cranial trigeminal ganglion (TG), which is located between the eye and the otic capsule in close proximity to the anterior lateral line ganglion. From there, their projections arborize and cover the entire skin with fine nerve endings that can sense pressure differences that impact the skin (Figure 1B). The extensive branching makes every part of the head touch-sensitive (Sagasti et al., 2005).

Similar to the lateral line, the auditory system relies on detecting pressure and displacement gradients in the surrounding medium by hair cells. Physically, there is no difference between sound and water flow. However, speed and the temporal structure of the water motion may differ depending on the frequency and intensity of the signal. Teleost fish have an inner ear consisting of otoliths and a series of hair cells. This system is used both for perceiving sound and acceleration (the vestibular system). The primary afferent neurons in the statoacoustic cranial ganglion are the first stage of processing sound information encoded by the auditory hair cells and forward signals to the torus semicircularis located in the hindbrain (Higgs and Radford, 2013; Privat et al., 2019).

Understanding the way in which the lateral line and the trigeminal neurons perceive water flow fields has broad implications for neurobiology and developmental biology, as well as for application-orientated fields like engineering and biomimetics. By unraveling the intricate sensory processes involved in flow field perception, researchers can gain insights into the design and development of bio-inspired underwater robots, sensors, and hydrodynamic systems. Therefore, in constructing underwater robots, it is a fruitful endeavor to find inspiration in the way aquatic animals perceive and navigate the surrounding environment since robots may encounter similar challenges as aquatic animals (Liu et al., 2016; Haehnel-Taguchi et al., 2018a). There is already a growing field of scientists and engineers trying to combine their knowledge to improve the construction of a variety of sensors and aquatic vehicles. Artificial flow sensors, for example, have been constructed based on micro-electro-mechanical systems (MEMS), and there are numerous approaches on how to design such systems (Tao and Yu, 2012; Liu et al., 2016; Jiang et al., 2019).

Although the development, anatomy, and function of the posterior lateral line system have been studied intensively, much less focus has been set on the anterior part of the lateral line. Compared to the posterior lateral line, the anterior part of this sensory system is more complex in terms of anatomical structure and, possibly, in physiological function as well. Instead of the mostly linear organization along the length of the body of the posterior lateral line, the anterior neuromasts are distributed over the entire surface of the head and are tuned to a variety of different flow directions (Chou et al., 2017). Therefore, we hypothesize that the anterior lateral is much more capable of encoding three-dimensional flow fields, helping the animal to get more information about the subtle changes of water flow around its head.

The temporal development and patterning of the anterior lateral line were described recently for the first time in detail (Iwasaki et al., 2020). We could confirm the order of neuromast development and the anatomical structure of the anterior lateral line. Here, we aim to characterize and classify the response repertoire of not only the anterior lateral line neurons but also the first stages of the trigeminal and auditory sensory system in larval zebrafish. We investigate different stimulus modalities, which we classify as tactile (Figures 1A, 3A), auditory (Figure 5A), or water current (Figure 6A). These findings will contribute to a better understanding of flow perception and inspire future generations of biomimetic sensors and underwater robots.

## 2 Materials and methods

### 2.1 Experimental animals and transgenic lines

The zebrafish (*Danio rerio*) is a freshwater fish (family: Cyprinidae) native to South Asia and a well-established model organism in developmental biology (Lele and Krone, 1996) and neuroscience (Briggs, 2002; Friedrich et al., 2010). The large variety of available genetic tools and transgenic lines, the translucent brain, the rich repertoire of behavior in early larval stages, and the relatively easy maintenance of adults and larvae make the zebrafish an ideal subject for studying the lateral line and other sensory systems on a cellular level (Wyatt et al., 2015).

Here, we used zebrafish larvae to study the physiological response patterns of different sensory neuron types to a set of stimulus modalities. Zebrafish were kept in our animal facility under standard conditions (Westerfield, 2000). For experiments, we bred and raised zebrafish larvae on a 14/10 h light/dark cycle at 28.5°C in E3 Medium. All larvae for the calcium imaging experiments were 4–5 dpf (days post fertilization) old and had a *mitfa*−/− (nacre) or Casper (mitfa w2, mpv17a9/a9) background to suppress skin pigmentation, which is essential for imaging neurons in the brain, because pigments would block or scatter the light source directed onto these neurons.

To record neural activity in the peripheral sensory ganglia, we used the transgenic lines Tg (UAS:GCaMP6s)^mpn101^ (Thiele et al., 2014) and Tg (isl2b:Gal4)^zc65^ (Fujimoto et al., 2011). These transgenic animals expressed the genetically encoded calcium indicator (GECI) GCaMP6s. The molecular structure of this indicator is derived by fusing a green fluorescent protein (GFP), calmodulin (CaM), and a peptide sequence called M13. Upon binding to calcium ions, GCaMP undergoes a conformational change that leads to an increased fluorescence of the GFP, thus signaling the current calcium concentration in the cell. Since this concentration is tightly connected to the activity of a neuron (generation of action potentials), the fluorescence intensity can be used as a proxy for neuronal activity.

In the anterior lateral line development experiments, we imaged Tg (HGn39D:GFP)^bc2Tg^ (Faucherre et al., 2009) and Tg (Brnc3:GFP)^s356t^ (Svoboda et al., 1996) larvae. The first line expressed GFP in all sensory afferent neurons of the lateral line. The second line expressed GFP in all hair cells of the lateral line and the audio-vestibular system. All experimental procedures were in accordance with German laws for animal care, and a permit was obtained from the Regierungspraesidium Freiburg.

### 2.2 Mechanosensory stimulation

Lateral line afferent, trigeminal, and auditory neurons have evolved to encode specific physical properties of the surrounding environment of the animal. The lateral line is tuned to water motion, the trigeminal system to touch, temperature, and noxious chemicals, and the auditory system to sound waves. To record responses of the anterior lateral line, trigeminal, and auditory afferent neurons and provide a repeatable and reliable stimulation, we used a mechanosensory stimulation procedure as previously described by Reinig et al. (2017). We used a platinum wire (0.127-mm diameter, Alfa Aesar) mounted to a holder that was attached to a piezo actuator (P-603.501, PI, Karlsruhe), which was controlled with custom written software in LabVIEW (National Instruments). The piezo actuator was then used to displace the platinum wire along one axis, precisely controlling speed and skin contact time. For stimulation, the platinum wire was placed directly in front of the larva’s head using a micromanipulator (Sensapex, SMX). At resting position, the wire was 100 µm away from the skin (Figures 1C, 3A). From there, it could move either at full speed (step duration: 100 and 400 ms) or at a preset constant speed (ramp velocity: 62.5, 125, 250, 500, and 1,000 μm/s). We presented a range of speeds to compute the neuron’s tuning curve to find the speed the neuron is most sensitive to. At the end of the movement, the platinum wire slightly impressed on the skin (Figure 1C). Due to the embedding in agarose, the thereby elicited displacement of the skin led to a deflection of the cupula of nearby head neuromasts while stimulating the skin pressure sensors (trigeminal nerve endings) as well. This method of stimulation proved to be much more reliable and feasible in driving primary sensory and secondary ascending neurons than water current applications. To prevent any bias by the stimulus-type sequence, stimuli were presented in a pseudo-randomized order. The calcium signal of each neuron was evaluated in a time window of 30 s (5 s before and 25 s after stimulus onset).

### 2.3 Sound stimulation

Sound is transmitted in water by waves of different pressure levels. These sound waves are detected by the auditory system and, depending on the carrier frequencies, by the lateral line as well (Higgs and Radford, 2013). To compare direct tactile stimulation with other types of stimuli, we played back the sound to the larva via a sound speaker. To achieve this, we used a small speaker (FRWS5, Visaton) that was attached to the stage surface onto which the dish containing the embedded larvae was placed. This way, the sound waves could travel through the plate into the dish and the fluid inside the dish, thereby reaching the embedded animal (Figure 5A). The speaker was connected to an audio amplifier (AMP2.2 LN 3W, Visaton), and a custom Python code was used to playback an audio signal. The carrier frequency was 2 kHz, and we generated 2-s-long pulses using a rectangular amplitude modulation of the carrier sine wave. A sound frequency of 2 kHz is high enough to be differentiable from the typically low-frequency components of the lateral line water flow regime but still audible for zebrafish larvae. Two single pulses with an interval of 15 s were followed by a train of 15 pulses with an inter-pulse interval of either 5 s or 10 s. The gap between the last single pulse and the first pulse of a train was always 60 s. Since calcium signals from GCaMP6s are relatively slow (a decay time of several seconds), it was necessary to have long enough inter-stimulus intervals so that the recorded signals were still discriminable.

To analyze the responses to the presented stimuli, the calcium signal of each neuron was evaluated in a predefined window. For single pulses, the window started 1 s before and ended 15 s after stimulus onset. For pulses belonging to a train (train pulses), the window was, due to the shorter inter-pulse intervals, 1 s before and 4 s after stimulus onset. Furthermore, we analyzed the entire pulse train on its own (5 s before and 200 s after the onset of the first pulse in the train).

### 2.4 Water flow stimulation

Although the tactile stimulation of the lateral line elicited strong responses, we additionally stimulated larvae with water current. To stimulate the anterior lateral line system of larval zebrafish, two glass pipettes with a diameter of 30 µm were connected to a syringe pump (Chemyx Fusion 200) to generate a laminar bidirectional water flow. One of the glass pipettes was positioned in front and slightly above the larva’s head. The second glass pipette was located behind the larva, and either one could be used as sink or source of the water flow (Figure 6A). In order to stimulate the neuromasts of the anterior lateral line, we carefully removed the agarose around the entire head. During experiments, the direction of the water flow was sequentially alternated between head-to-tail (HT) and tail-to-head (TH), and the flow speed was increased from 1 mL/min to 5 mL/min. Each water jet was 10 s long, with a 25-s pause before the next stimulus was presented.

### 2.5 Two-photon calcium imaging and analysis

When neurons are active and generate action potentials (spikes), there is an increase in intracellular calcium ions that enter the cell via voltage-gated calcium channels. This correlation between calcium concentration and neuronal activity is exploited by using calcium indicators that change fluorescence intensity by binding to calcium inside the cell (Kettunen, 2020). Calcium imaging, therefore, allowed us to record the activity of several neurons simultaneously in one animal.

For calcium imaging experiments, larvae (4 dpf old) were anesthetized with 0.02% tricaine (ethyl 3-aminobenzoate methanesulfonate; Sigma, St. Louis, MO) and bath-treated with α-bungarotoxin (10 µL of a 0.1% solution, *Bungarus multicinctus*, Calbiochem) for 10–15 min to immobilize the larvae, thus avoiding any imaging artifacts due to motion. Then, the larvae were transferred to a Petri dish with E3 to stop the incubation, where they could be screened for lack of motion and normal physiological parameters (e.g., heart beat and blood flow). Subsequently, the larvae were mounted upright in 1.6% low melting agarose to fixate them, allowing for long recordings under the microscope. For tactile stimulation of the forehead, a small window in front of the head was cut into the agarose, such that the platinum wire could access the forehead skin. Afterward, the larvae were left to recover for 30 min before starting the experiments.

To start an experiment, paralyzed larvae were placed on a stage under a movable objective microscope (MOM, Sutter Instrument) equipped with a 2-photon laser (Coherent). The laser was used to excite GCaMP6s, which is needed to elicit fluorescence of the fused GFP. The fluorescence light is then detected by photomultiplier tubes (PMTs) and stored on a computer. Larvae expressing GCaMP6s in the peripheral sensory neurons were imaged with a frame rate of 2 fps and a wavelength of 920 nm with approximately 20 mW power at the sample. For every animal, a z-plane with the maximal number of neurons expressing GCaMP6s was recorded. To correct for movement artifacts, the recordings were registered to a standard deviation projection of 400 frames with the least movement using the TurboReg Plugin (Thévenaz et al., 1998) in ImageJ (NIH). Recordings with non-uniform movement artifacts were registered using the non-rigid registration method included in the Python package suite2p (Patriarchi et al., 2018). After registration, regions of interest (ROIs) were drawn manually around neurons, guided by a standard deviation projection of the calcium recording. ROI fluorescence intensity was extracted as the mean intensity of all pixels included in that ROI. The raw fluorescence time series were converted to ∆F/F:

$$\frac{\Delta F}{F_{0}}=\frac{F\left(t\right)-F_{0}}{F_{0}}.$$

The baseline fluorescence F_0_ was calculated by taking the 5th percentile value of the entire trace. This normalization step is necessary since calcium indicators like GCaMP can exhibit fluctuations in baseline fluorescence intensity due to changes in indicator concentration, uneven distribution, and photobleaching. By calculating the relative change in fluorescence (ΔF) compared to the baseline fluorescence (F), ΔF/F normalizes the signal to account for these baseline variations. For an unbiased comparison of neural activities of different cell types and between animals, ∆F/F traces were transformed to *z*-scores ( µ : mean ∆F/F; σ : standard deviation of ∆F/F):

$$z=\frac{\frac{\Delta F}{F_{0}}-µ}{\sigma }.$$

Instead of sorting cells manually and to avoid biased selection of responsive and non-responsive neurons, cells were detected automatically by using a linear regression model approach (Miri et al., 2011; Helmbrecht et al., 2018; Förster et al., 2020). To compute the regressors, first, the stimulus (voltage trace) of each recording was converted to a binary trace with only ones (stimulus on) and zeros (stimulus off), yielding a rectangular function over time. This function has either a value of one, corresponding to time points when the stimulus was on, or zero, when there was no stimulus present. This binary function was then convolved with an ideal calcium response function (ICRF), representing an ideal calcium transient (response) of an ideal neuron:

$$ICRF=e^{-\frac{t}{\tau }}.$$

Since we do not have access to the ideal neuron, the exponential time constant τ, describing the decay of fluorescence response, was determined by fitting the ICRF to all calcium responses in all recordings using a non-linear least squares method (Python scipy.optimize.curve_fit function). Every recorded neuron (ROI) was then scored by computing an ordinary least squares linear regression model (Python scikit-learn sklearn.linear_model.LinearRegression) between the neuron’s ∆F/F trace (y) and the corresponding regressor (x):

y=a+bx.

With ***a*** as the intercept and ***b*** as the slope (coefficient), the final score was then computed by multiplying the coefficient of determination (R^2^) with the slope ***a***. This yielded a single value that described how strongly a neuron responded to a specific type of stimulation. All neurons with a score below 0.1 were discarded.

For all data analysis and visualization, we used custom written code either in MATLAB (MathWorks) or Python. Classification of functional subtypes of trigeminal neurons was performed using a fixed threshold value of 0.5 SD (mean *z*-scored response to ramps). This means if the mean response of a neuron to ramp stimulation was above 0.5 SD away from its baseline activity, we considered this neuron to be responsive.

### 2.6 Anterior lateral line neuromast development

In order to cover many stages in the larval development over 1 week, three groups of zebrafish larvae were raised under different temperature regimes at 28.5°C (standard development) and at 26.5°C, to slow down the standard development, according to the linear relationship:

$$H\left[t\right]=\frac{h}{0.055\;T-0.57},$$

where H [t] is the developmental stage in hours defined by temperature T and h in hours after fertilization at 28.5°C incubation temperature (Kimmel et al., 1995). After 17 hpf (hours post fertilization), eggs were dechorionated after incubation with Pronase (200 µL of Pronase in 20 mL E3) for 10–15 min. To stop the enzymatic function of Pronase, BSA (1% bovine serum albumin) was added, and eggs were washed in E3 four times. At an age of 21.5 hpf, larvae were treated with PTU (0.2 M propylthiouracil) to suppress pigmentation.

To image the developmental stages of neuromasts, larvae were anesthetized with 0.02% tricaine and mounted in 1.6% low-melting agarose. For epi-fluorescence imaging, we used an Axio Examiner Stereo microscope (Zeiss, Oberkochen, Germany) equipped with a camera, and images were captured using manufacturer software ZEN pro (Zeiss). The z-stack step and stack size were adjusted to incorporate all visible neuromasts. During development, zebrafish embryos elongate the anterior–posterior body axis, thus stretching the initially bent trunk and tail. To not disturb this elongation process, larvae were taken out of the agarose and placed in E3 medium overnight.

Z-stack planes showing clearly visible neuromasts (hair cells) were projected into a single plane using Adobe Photoshop to show all neuromasts of one developmental stage, and brightness and contrast were adapted to increase the visibility of maturing hair cells.

### 2.7 Statistics

To identify significant differences in response types between neuron types and stimulation sets, we used ordinary least squares linear models (Python, statsmodels.regression.linear_model.OLS) and ANOVA (Python, statsmodels.stats.anova.anova_lm) based on such models. From these models, we extracted the corresponding R^2^, F-statistics, and *p*-values.

## 3 Results

### 3.1 Temporal development of anterior lateral line neuromasts

**FIGURE 2:**
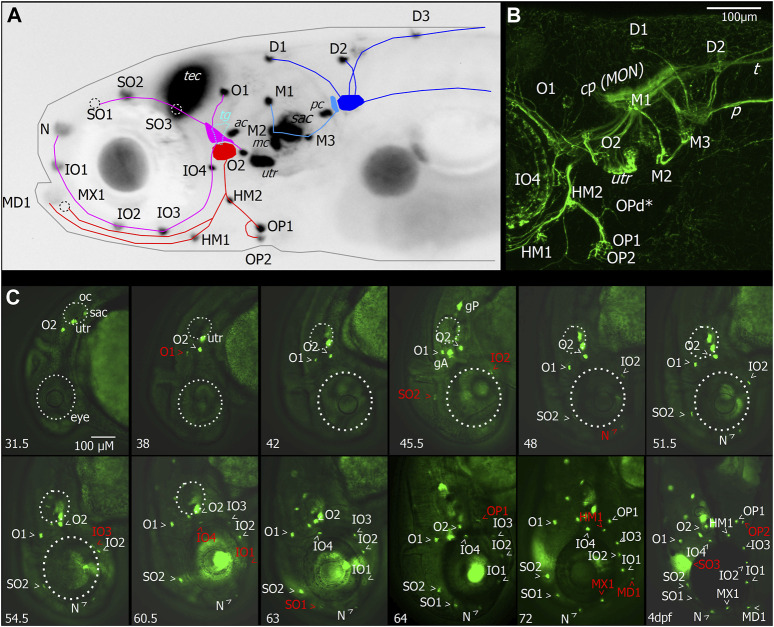
Layout and temporal development of anterior neuromasts in larval zebrafish. **(A)** Micrograph of the head of a 7dpf larva expressing GFP under the Brn3c promotor in hair cells, tectum (TEC), and lens with superimposed schematic representation of the neuromast layout and innervation derived from imaging of afferent neurons, as shown in **(B)**. The supraorbital (SO1–3) and otic neuromasts (O1–2), as well as infraorbital neuromasts (IO1–4) and nasal neuromasts, comprise the anterodorsal lateral line (magenta). They are innervated by four nerve branches arising from the anterodorsal ganglion. The anteroventral lateral line system (red) comprises the mandibular (MD1), hyomandibular (HM1–2), and opercular (OP1–2) neuromasts. These neuromasts are innervated by nerve branches originating from the anteroventral ganglion. When viewed from the side, the trigeminal ganglion (TG) is located between the anterior lateral line ganglia (cyan). Neuromasts also located on or in proximity of the head which are part of the posterior lateral line are the medial neuromasts (M1–3) innervated by neurons arising from the medial ganglion (light blue). The dorsal neuromasts (D1–3) are innervated by the dorsal branch of the posterior lateral line arising from the posterior lateral line ganglion (blue). Also labeled with GFP are hair cells of the sensory epithelia of the statoacoustic system, the anterior and posterior cristae (AC and PC), the anterior macula (MC), utricle (UTR), and sacculus (SAC). **(B)** Maximum intensity projection of confocal stack of the lateral view of the region around the lateral line ganglia with neurons of the cranial ganglia expressing YFP driven by islet2B in a 7 dpf larva. Innervation of the otic neuromasts, IO4, HM1, and opercular neuromasts is visible. In addition to the neuromasts visible in **(A)**, the opercular dorsal (OPd) neuromast can also be observed (asterisk). Nerve branches of the posterior lateral line, the posterior lateral line nerve (P), and innervation of the skin by trigeminal neurons and the trigeminal nerve projecting caudally (T) are also visible. **(C)** Micrographs depicting the temporal patterning of neuromasts in the anterior lateral line system in larvae expressing GFP in hair cells driven by Brn3c. Age of imaged larvae is given in the bottom left corner in hours post fertilization (hpf) and finally at 4dpf. Abbreviations as in **(A)**. Larva imaged at 45.5 hpf also expressed GFP driven by *sill* in afferent neurons of the anterior and posterior lateral line ganglia (ALLG and PLLG). Later stages are still represented with head pointing down for comparison although head position relative to body axis has changed. Outline of the eye and otic capsule is indicated by dashed white lines up to 54.5 and 60.5 hpf for orientation.

The neuromasts of the anterior lateral line (ALL) are distributed in a stereotypical pattern on the head, just as the neuromasts of the posterior lateral line (PLL) are distributed stereotypically on the trunk. We imaged the developing ALL using transgenic lines Tg (HGn39D:GFP)^bc2Tg^, also referred to as *sill*:GFP (sensory innervation of the lateral line), which labels the primary sensory afferent neurons of the lateral line; Tg (Brnc3:GFP)^s356t^, which labels hair cells (Figures 2A, C); and Tg (isl2b:Gal4)^zc65^, which labels many cranial sensory neurons (Figure 2B). The ALL originates from two ALL placodes: the anterodorsal (AD) and anteroventral (AV) placodes (Iwasaki et al., 2020). The neuromasts derived from the AD placode are the supraorbital (SO1–3) and otic neuromasts (O1–2), as well as infraorbital neuromasts (IO1–4) and nasal (N) neuromasts (Figure 2A). Four nerve branches innervate the AD ALL neuromasts, arising from the AD ganglion: the superior otic ramus innervating O1, the inferior otic ramus innervating O2, the superior ophthalmic ramus innervating SO1–3, and the buccal ramus innervating N and IO1–4 (Figure 2B).

The AV ALL system comprises the mandibular (MD1), hyomandibular (HM1–2), and opercular (OP1–2) neuromasts (Figure 2A). These neuromasts are innervated by afferent nerves originating from the AV ganglion extending ventrally in a single fascicle and later splitting into rami innervating OP1–2 and d, HM1 and MD1, and MX1 (Figures 2A, B). When viewed from the side, the trigeminal ganglion (TG) is located between the ALL ganglia, and trigeminal neurons extend into the periphery innervating the skin (Figure 2B). Neuromasts which are part of PLL are the medial neuromasts (M1–3) innervated by neurons arising from the medial ganglion, and dorsal neuromasts (D1–3) are innervated by the dorsal branch of the PLL arising from the posterior lateral line ganglion (Figures 2A, B). Also labeled with GFP (Figure 2A) are hair cells of the sensory epithelia of the statoacoustic system, the anterior and posterior cristae (AC and PC), the anterior macula (MC), utricle (UTR), and sacculus (SAC).

To investigate the temporal dynamic of neuromast patterning, we performed time-lapse imaging in larvae expressing GFP in hair cells and primary afferent neurons. At 31.5 h, we first observed O2 derived from the AD ALL, followed by O1 at 38 hpf (Figure 2C). Since these neuromasts appear in the position of the placode, they are considered “homegrown” (Iwasaki et al., 2020). At 45.5 hpf, we first observed SO2 and IO2 derived from the AD and considered “migratory” as they are deposited by the migrating primordium. At 48 hpf, N first appears as the most distal neuromast, probably budding from IO2 (Iwasaki et al., 2020). At 54.5 hpf, we first observed IO3 considering “intercalary” differentiating between two already deposited neuromasts. At 60.5 hpf, we observed IO1 budding from IO2 and IO4 as intercalary neuromast. At 63 hpf, we first observed SO1, budding from SO2. At 64 hpf, we observed OP1, the first neuromast derived from AV, which, in some individuals, was already observed at 63 hpf (Table 1), budding from the HM primordium OP1. At 72 hpf, we observed MD1 and MX1 derived from AV, which have also been described to bud from the HM primordium (Iwasaki et al., 2020), and HM1 is deposited. At 4 dpf, we observed SO3 developing intercalary between SO2, and O1 and OP2 budding from HM1. A summary of ALL development up to day 4 is given in Table 1. OPd only appears at a much later time point; however, we already observed faint GFP expression in the innervation of OPd at 7 dpf (Figure 2B). We next wanted to investigate if the anterior lateral line is functional at larval stages (4 dpf) and how the anterior lateral line afferent neurons that receive inputs from the anterior neuromasts encode stimuli.

**TABLE 1:** Temporal patterning of neuromasts [^a^ observed at later time points in some individuals; ^b^ only innervation observed (compare Figure 2B)].

Neuromast		Time point first observed (hpf)											
		31.5	38	45.5	48	50.5	54.5	60.5	63	64	72	4dpf	7dpf
Anterior dorsal	*Otic*	O2	O1										
	*Supraorbital*			SO2					SO1			SO3	
	*Infraorbital*			IO2		IO4^a^	IO3	IO1					
	*Nasal*				N								
Anterior ventral	*Opercular*								OP1^a^			OP2	OPd^b^
	*Hyomandibular*										HM1		HM2
	*Mandibular*										MD1		
	*Maxillary*										MX		

^a^ observed at later time points in some individuals.

^b^ only innervation observed (compare Figure 2B).

### 3.2 Afferent neurons of the anterior lateral line and trigeminal ganglia respond to mechanosensory stimulation

**FIGURE 3:**
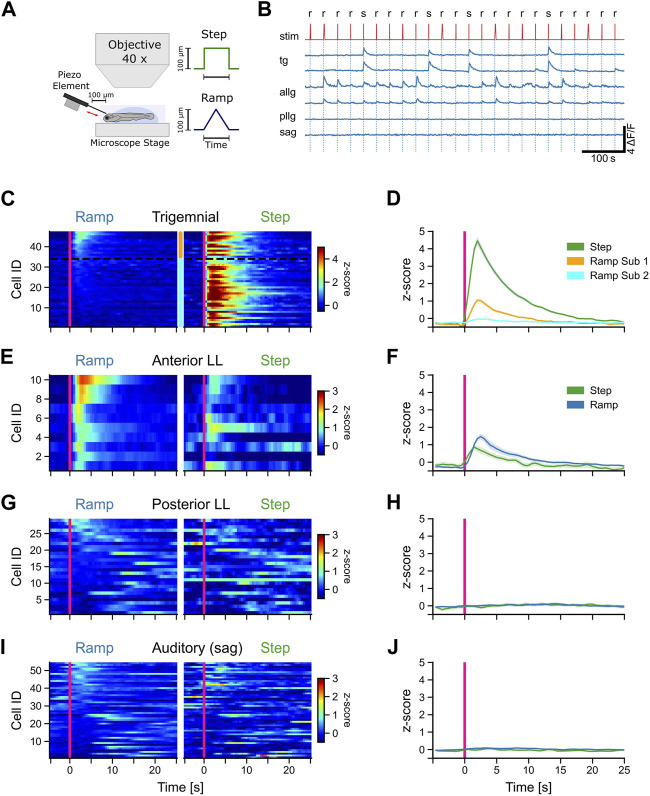
Afferent trigeminal and anterior lateral line neurons respond to tactile stimulation. **(A)** Schematic representation of the experimental setup and the stimulus shapes (step and ramp) used in the experiments. **(B)** Representative calcium traces (dF/F) of selected trigeminal neurons (TG), anterior lateral line neurons (ALLG), posterior lateral line neurons (PLLG), and auditory ganglion neurons (SAG). Stimulus trace (stim) is shown in red with ramp (r) and step (s). **(C, E, G, I)** Mean activity (z-score) of individual neurons to ramp and step stimulation for TG neurons **(C)**, ALLG neurons **(E)**, PLLG neurons **(G)**, and auditory neurons in the SAG **(I)**. Stimulus onset at *t* = 0 s indicated by a pink vertical line. Z-score values over time are color-coded, as represented in the scale bar to the left. The *y*-axis shows the IDs of the individual neurons, sorted from top to bottom starting with the highest activity during ramp stimulation (mean activity from stimulus onset to 10 s after stimulus onset). The two subtypes of trigeminal neurons in **(C)** are indicated by the orange (subtype 1) and cyan (subtype 2) bars between the stimulus-type blocks and separated by a black dotted line. **(D, F, H, J)** Mean activity (z-score) of all neurons of a given cell type (solid lines). The shaded area represents the standard error of the mean (SEM). The pink vertical line indicates the stimulus onset. Trigeminal subtype 1 (*n* = 13), trigeminal subtype 2 (*n* = 34), anterior lateral line (*n* = 10), posterior lateral line (*n* = 29), and auditory ganglion (*n* = 54).

While navigating underwater, fish rely on a diverse set of continuously arriving sensory inputs. Alongside visual sensations, mechanosensory stimulation is especially important since fish must be able to orient in darkness as well. To investigate the response patterns of sensory afferent neurons to tactile stimulation, we recorded calcium signals from 4 dpf old transgenic larval zebrafish [Tg (isl2b:Gal4)^zc65^] using the genetically encoded calcium indicator GCaMP6s [Tg (UAS:GCaMP6s)^mpn101^] and two-photon microscopy from afferent neurons located in the peripheral sensory ganglia (anterior lateral line, trigeminal, posterior lateral line, and statoacoustic ganglia). Embedded larvae were mechanically stimulated with both steps (maximum speed) and ramps (predefined slower speed) using a platinum wire that approached and touched the larva’s forehead (Figure 3A). The order of steps and ramps was pseudo-randomized, and between each stimulus presentation, there was an interval of 30 s (Figure 3B).

All trigeminal neurons responded strongly to steps but showed different responses to stimulation with ramps [F (1, 262) = 823, *p* < 0.001]. One group of trigeminal cells was exclusively excited by steps. One example is shown in the upper trigeminal trace in Figure 3B. Each stimulation elicited a strong rise in the calcium signal (*z*-scores larger than 3 SD). However, there were also some neurons that showed a distinct, albeit weaker (*z*-scores around 1 SD), response to ramp stimulation (lower trigeminal trace in Figure 3B). These two response patterns are evidence for distinct functional subtypes of trigeminal neurons that differ in their tuning to stimulus speed and contact duration or intensity. Based on these response differences, we subdivided the trigeminal neurons into two subgroups. Classification was performed by using a fixed threshold of 0.5 standard deviations for the *z*-scored mean activity values of each cell in a window from stimulus onset to 10 s afterward. Every neuron that showed a score above this threshold was considered to belong to subgroup 1 (Figures 3C, D). Out of 47 recorded cells, 13 were classified as subtype 1 and 34 as subtype 2. Neurons belonging to one subtype showed significantly different responses compared to ramps to neurons of the other subtype [F (1, 262) = 24, *p* < 0.001]. The clustering was, however, purely functional since we could not find any differences in anatomical mapping or overall morphological appearance in the recordings. Therefore, we concluded that there are two distinct functional types of trigeminal neurons in zebrafish.

In contrast to the trigeminal afferent neurons, anterior lateral line ganglion cells showed a preference for ramp over step stimulation, although all of them responded to steps as well. However, responses to steps and slow ramps were weaker than responses to fast ramps (Figures 3B, E, F). The mean *z*-score trace for steps showed an earlier rise time than that for ramps, mostly because three out of ten neurons had substantial spontaneous activity right before stimulus onset (Figures 3E, F: step), which led to a slight shift in the response onset time. Anterior lateral line activity during ramps and steps was comparable in strength to the activity of trigeminal subtype 1 neurons during ramp stimulation. In general, trigeminal peak responses to steps were five-fold larger.

We also recorded all other cranial ganglia cells that were labeled by the Tg (isl2b:Gal4) driver line. However, none of those were responsive to tactile stimulation of the forehead. Posterior lateral line afferent neurons, which innervate neuromasts along the trunk, did not respond to any tactile stimulation of the head (Figure 3H) but showed small spontaneous fluctuations of the calcium traces (spontaneous activity) during the recording session (Figures 3G, H). This suggests that the stimulation method we used is strictly restricted to the head only since the anterior lateral line was activated during stimulation but not the posterior lateral line.

Similarly, auditory afferent neurons in the statoacoustic ganglion (SAG) were unresponsive to tactile stimulation, as expected, but spontaneously active (Figures 3I, J). In summary, tactile stimulation of the head is encoded by two different sensory systems, the anterior lateral line and the trigeminal system, with no contribution by the posterior lateral line or the auditory neurons in the statoacoustic ganglion.

### 3.3 Anterior lateral line and trigeminal neurons have different velocity tunings

**FIGURE 4:**
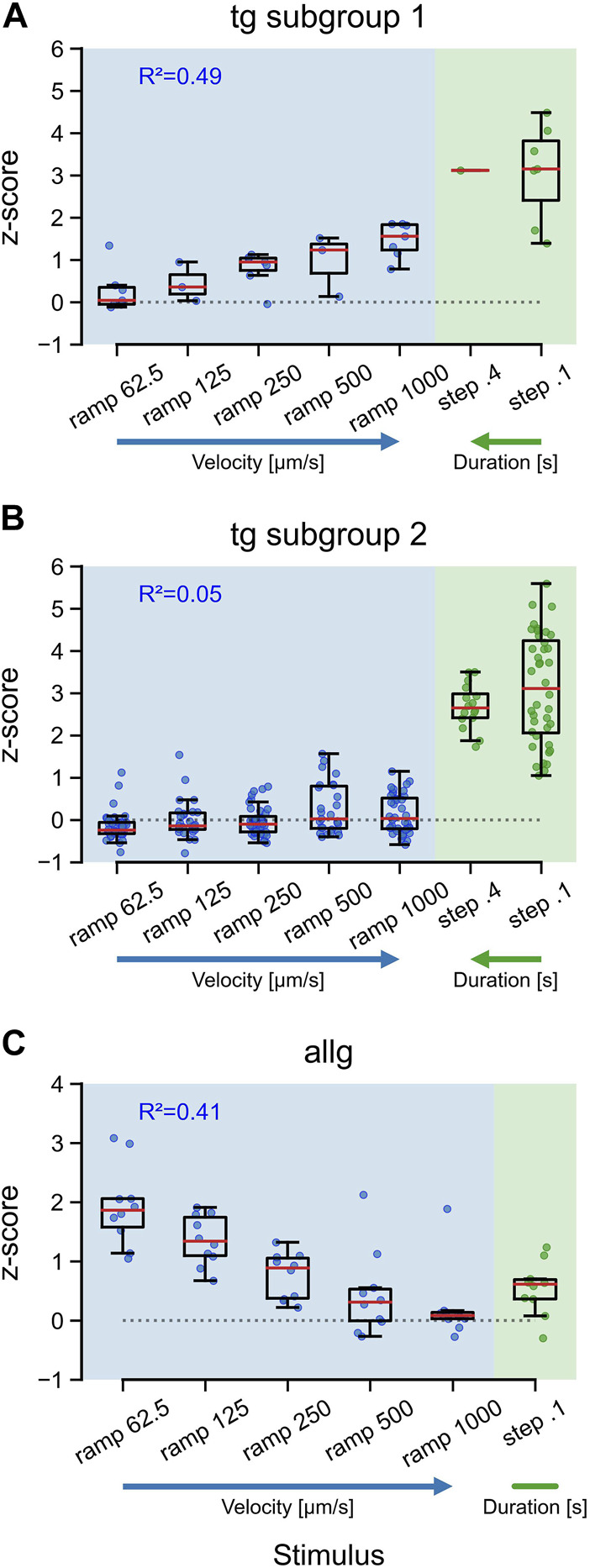
Tuning of trigeminal and anterior lateral line neurons to the velocity and duration of tactile stimulation. Boxplots for responses (*z*-score) of trigeminal subtype 1 neurons **(A)**, trigeminal subtypes 2 neurons **(B)**, and anterior lateral line neurons **(C)** to ramp and step stimulation. Blue and green dots indicate the mean activity of individual neurons between the stimulus onset and 15 s after stimulus onset. Red lines represent the median values. Horizontal dotted lines indicate *z* = 0. R^2^ values are for a linear model including only the ramp velocities. Ramp stimuli are given in velocity [µm/s] increasing from left to right and step stimuli in duration [s] increasing from right to left, as further indicated by the blue (velocity) and green (duration) arrows. Trigeminal subtype 1 (from left to right): n_62.5_ = 7; n_125_ = 3; n_250_ = 7; n_500_ = 3; n_1000_ = 7; n_0.4_ = 1; n_0.1_ = 7. Trigeminal subtype 2 (from left to right): n_62.5_ = 40; n_125_ = 27; n_250_ = 40; n_500_ = 27; n_1000_ = 40; n_0.4_ = 16; n_0.1_ = 40. Anterior lateral line for all: *n* = 10.

Natural stimuli are rarely of a constant, unchanging type. Intensity and velocity may be quite different over time, and survival can depend upon reliably encoding those changes. To evaluate if afferent neurons show specific tunings to different velocities and durations of tactile stimuli, we used a range of velocities for ramps (62.5, 125, 250, 500, and 1,000 μm/s) and two duration times for steps (0.1 and 0.4 s). Both trigeminal subgroups responded maximally to steps, independent of the duration [TG subgroup 2: F (1, 54) = 1.68, *p* = 0.2], with a median activity of approximately 3 SD (Figures 4A, B: steps). Trigeminal subgroup 2 neurons were not responding to ramps, and therefore, the median activity stayed around baseline (Figure 4B), independent of velocity (R^2^ = 0.05, *p* < 0.01). Subtype 1 neurons, however, showed a clear trend (R^2^ = 0.49, *p* < 0.001) in their tuning to high velocities for ramps (Figure 4A). For anterior lateral line neurons, this tuning was reversed (R^2^ = 0.41, *p* < 0.001). Slow ramps elicited much stronger responses than fast ramps, including the very fast step stimuli (Figure 4C). Interestingly, both trigeminal subgroups displayed maximal responses to steps, regardless of the duration, suggesting that the trigeminal system is primarily sensitive to the presence or absence of tactile stimuli rather than their duration. Additionally, trigeminal subgroup 2 neurons exhibited almost no responses to ramps, indicating a lack of sensitivity to changes in velocity. This finding suggests that trigeminal subgroup 2 neurons may be more specialized for detecting static or slowly varying tactile stimuli, which could be particularly relevant for tasks involving object manipulation or fine touch discrimination. Intriguingly, subtype 1 neurons of the trigeminal group demonstrated a clear trend of velocity tuning for ramps, preferring higher velocities. This characteristic may reflect their role in rapidly detecting and encoding tactile stimuli associated with fast movements or dynamic interactions with the environment.

### 3.4 Sound stimulation exclusively elicits responses in afferent auditory neurons

**FIGURE 5:**
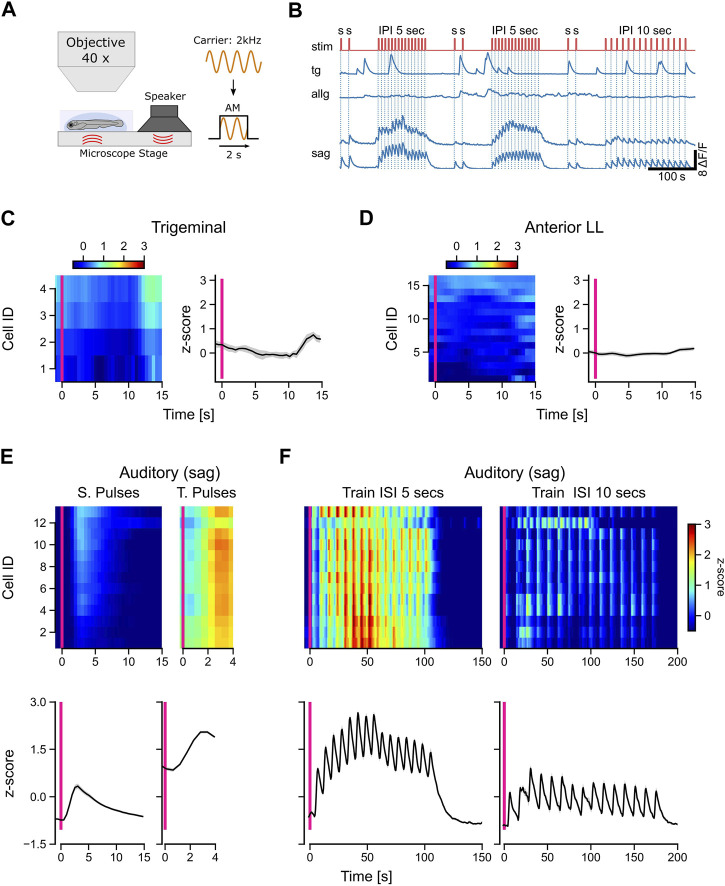
Only auditory neurons respond to acoustic stimulation. **(A)** Schematic representation of the experimental setup and the stimulus used in the experiments. A 2-kHz carrier sine wave was modulated with a step function to turn sound stimulation on and off, thereby generating pulsed tones with a duration of 2 s. **(B)** Calcium traces (dF/F) of selected trigeminal neurons (TG), anterior lateral line neurons (ALLG), and auditory ganglion neurons (SAG). The stimulus trace is shown in red with single pulses (s) with a duration of 2 s and pulse trains with 15 pulses with a pulse duration of 2 s and an interval of either 5 s or 10 s between the pulses. **(C, D)** Left in each panel: mean activity (*z*-score) of individual trigeminal **(C)** and anterior lateral line **(D)** neurons to single sound pulses. The *y*-axis shows the IDs of the individual neurons, which are sorted from top to bottom starting with the highest activity during stimulation (10 s after the stimulus onset). Right in each panel: mean activity (*z*-score) of all trigeminal **(C)** and all anterior lateral line **(D)** neurons (*n*: 4 and 16, respectively). **(E, F)** Top in each panel: Mean activity (*z*-score) of individual auditory ganglion neurons (*n* = 14) to single pulses [**(E)**, left], train pulses [**(E)**, right], and entire trains with inter-pulse intervals (ISI) of 5 s [**(F)**, left] and 10 s [**(F)**, right]. Bottom in each panel: mean activity (*z*-score) of all auditory ganglion neurons (SAG). The solid black line in [**(C, D)**, right] and [**(E, F)**, bottom] represents the mean response of all neurons; shaded region represents SEM. The pink vertical bar indicates stimulus onset in all panels. Activity (*z*-scores) in [**(C, D)**, left] and [**(E, F)**, top] is color-coded, as shown in the scale bars.

The source of tactile stimulation is always located in close proximity to the fish, whereas sound waves, for example, can travel much further through the water, reaching the fish even from far away. To have another stimulus modality in addition to the tactile stimulation, we used amplitude-modulated sine waves with a carrier frequency of 2 kHz. This pure tone was transmitted over a speaker to the ground plate so that larvae were stimulated by vibrations and airborne sound simultaneously (Figure 5A). We presented isolated rectangular sound pulses, followed by trains of 15 pulses with either 5-s or 10-s intervals (Figure 5B: stim).

Interestingly, neither trigeminal nor anterior lateral line afferent neurons responded to the sound pulses (Figures 5C, D). In contrast to the anterior lateral line cells, trigeminal neurons showed the typical strong spontaneous calcium peaks, which we observed in most of our recordings (Figure 5B). All auditory neurons (SAG) were activated during sound stimulation and followed strictly the temporal pattern of the pulse trains (Figure 5F). Single pulses evoked typical exponential calcium transients, while pulse trains showed a sustained activity during the entire stimulation period (Figure 5E). For inter-pulse intervals of 5 s, the responses were uniform across all cells, compared to the longer 10-s intervals (Figure 5F). Our findings highlight the selectivity of afferent auditory neurons for short wavelengths such as 2 kHz. In contrast, trigeminal and anterior lateral line neurons did not exhibit any discernible response to the sound pulses used in our experiment, indicating their specialization for processing other types of sensory input. The unresponsiveness of lateral line neurons further supports their specific sensitivity to lower frequencies.

### 3.5 Anterior lateral line neurons respond to water flow stimulation with directional sensitivity

**FIGURE 6:**
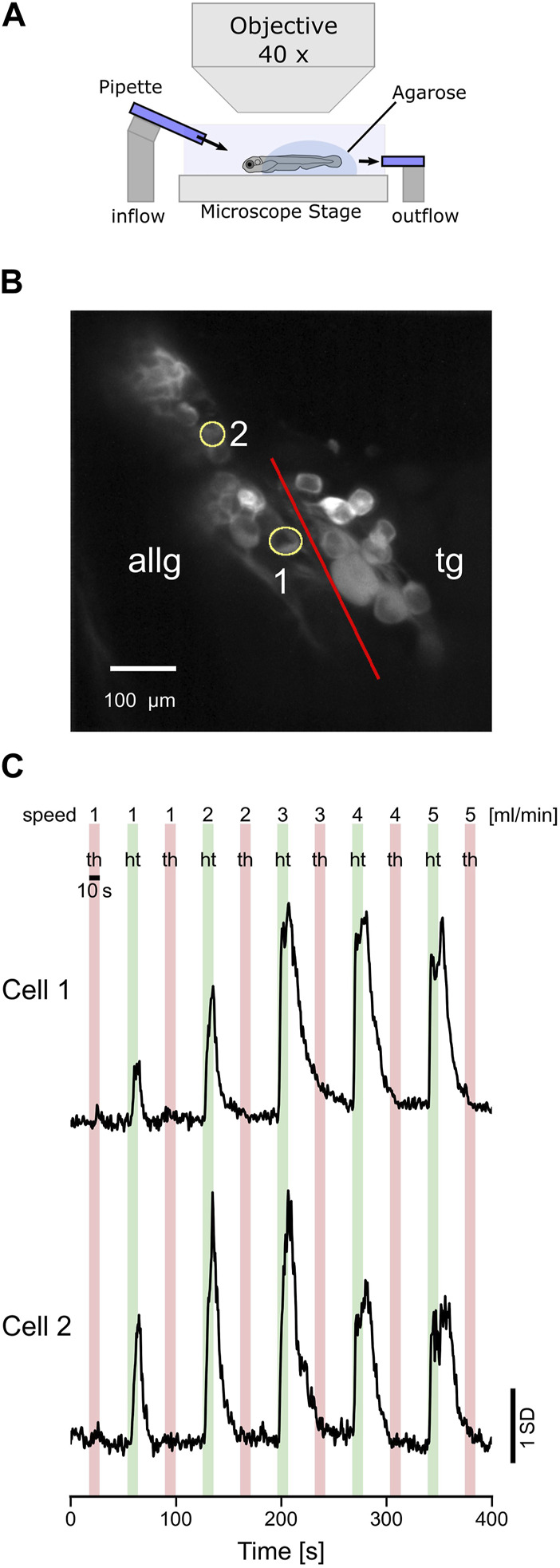
Afferent anterior lateral line neurons respond to water flow stimulation. **(A)** Schematic representation of the experimental setup and the stimulus used in the experiments. Two glass pipettes (diameter 30 µm) deliver laminar water jets in either head-to-tail (HT) or tail-to-head direction (TH). **(B)** Standard deviation of intensity projection of calcium imaging recording showing the neurons of the anterior lateral line ganglion (ALLG) and the trigeminal ganglion (TG). The red line indicates the border between those two ganglia. ROI numbers 1 and 2 (yellow circles) correspond to recordings shown in **(C)**. **(C)** Calcium traces (*z*-score) of two anterior lateral line ganglion neurons responding to head-to-tail (HT: green bars) stimulation but not to tail-to-head (TH: red bars) stimulation.

The biologically adequate stimulus for the lateral line is a change in water flow induced, for example, by conspecifics, prey, or predators. Therefore, we stimulated the anterior lateral line neuromasts with a water jet of 10 s in the head-to-tail and tail-to-head directions (Figure 6A). In total, we could only identify two afferent neurons that showed a clear response pattern in the anterior lateral line ganglion (Figure 6B). However, none of the trigeminal neurons were responsive in any way (data not shown). Possibly, the impact of the water current on the skin was not strong enough to elicit any responses in trigeminal neurons. Overall, water flow may stay in the subthreshold regime of trigeminal neurons. Furthermore, laminar water flow will lead to a global stimulation of the entire forehead skin. Eventually, individual trigeminal neurons with receptive fields covering the area of their respective nerve endings innervating the skin will get excited altogether. However, due to some putative inhibitory interconnections, neighboring cells will inhibit each other; thus, in total, no trigeminal neuron shows any activity. Both anterior lateral line neurons were direction-selective since they were only active during head-to-tail water flow. The same direction selectivity is well-known in the posterior lateral line hair cells and afferent neurons, and the anterior lateral line is, in its basic cellular units, not different from the posterior lateral line. Furthermore, cell 1 increased its activity with stimulus velocity, while cell 2 decreased its activity for the two fastest flow stimuli (4 and 5 mL/min) again (Figure 6C). In summary, these experiments show that anterior lateral line afferent neurons, similar to the posterior lateral line neurons, exhibit direction selectivity and tuning to different water flow speeds, comparable to the results we found for the tactile stimulation (see 3.3).

## 4 Discussion

Aquatic animals like zebrafish need to encode sensory information perceived from the underwater environment in which they live. Especially changes in speed and direction of either water flow or sudden tactile impacts on the surface of the animal’s body due to predators or prey pose an important challenge to the nervous system of aquatic animals. To advance the understanding of how such information is detected and processed by the sensory system, we analyzed the responses of peripheral afferent neurons in larval zebrafish to different stimulus modalities, which could potentially be encountered by fish in their aquatic habitat. We concentrated on the auditory, trigeminal, and anterior and posterior lateral line systems. The anterior lateral line is composed of hair cells arranged in neuromasts that are positioned in an elaborate way around the head. Several studies have described the development of the posterior lateral line, which develops from migrating primordia in a series of developmental waves (Raible and Kruse, 2000; Sapède et al., 2002; Nuñez et al., 2009; Sarrazin et al., 2010; Haehnel et al., 2012). However, there has been less focus on the anterior lateral line in the past. A recent study described how tissue interactions shape the development of the anterior lateral line and provided a comprehensive description of its developmental sequence and anatomy for larval and juvenile zebrafish (Iwasaki et al., 2020). We investigated the temporal patterning of neuromast positioning by time-lapse imaging (Figure 2C) and could confirm the sequence of their appearance. We then were interested in how tactile and flow stimuli in the zebrafish head are encoded, especially by the anterior lateral line, and how it might be different from stimuli encoding by the posterior lateral line system. There is evidence, for example, that the anterior lateral line is targeted in a specific way by efferent innervations (Manuel et al., 2021). The anterior lateral line also appears to differ from the posterior lateral line regarding the distribution of synapses of its projections in the hindbrain (Valera et al., 2021) and may play a distinct role in behavior (Groneberg et al., 2015).

By applying tactile stimulation, we observed distinct response profiles for the anterior lateral line and trigeminal afferent neurons. Afferent auditory and posterior lateral line neurons, however, do not respond at all to this kind of stimulation. This underlines that the presented tactile stimulation is purely mechanical and localized to the head only. For comparison, we also used sound stimuli and could find correlated activity only in auditory neurons, as expected. Furthermore, we found two functionally different subtypes of trigeminal neurons. The first subtype is exclusively activated by fast step stimuli, whereas the second subtype shows responses to the slower ramps as well. However, we could not find any connection to anatomical location or morphology of the functional diverse subgroups of trigeminal neurons. It has been shown that certain subtypes of trigeminal neurons play a role in thermoregulation and perception of noxious chemicals like capsaicin (Gau et al., 2013; Haesemeyer et al., 2018; Haesemeyer, 2020). However, to the best of our knowledge, this is the first time that functionally distinct mechanosensory subtypes in the zebrafish have been described in the literature. Our findings are comparable to the results of experiments performed on *Xenopus* tadpoles, where two differently responding sensory trigeminal neuron types were identified. In the tadpole, one subtype was described as “rapid-transient,” responding with just a few action potentials to fast stimulation. The other subtype was considered a “movement detector,” with tonic firing in response to slowly approaching stimuli (Roberts, 1980). It may be reasonable to consider the trigeminal subgroup 1, with its broader response profile to different velocities, as encoding some aspects of movement as well. However, more detailed experiments with either faster GCaMP variants or electrophysiology are needed to make more precise statements about this here. Furthermore, we noticed strong spontaneous calcium transients in many recordings of trigeminal afferent neurons (Figure 5B). The source and function of this activity are unclear. Either it could be intrinsic and driven by a pattern generator mechanism or it may be the result of efferent input from higher brain areas. One candidate for this may be the diencephalic dopaminergic clusters in the posterior tuberculum since those neurons innervate not only the lateral line ganglia but the trigeminal ganglion as well (Haehnel-Taguchi et al., 2018b).

Interestingly, anterior lateral line and trigeminal neurons have opposing velocity tuning curves. The former prefers slow velocities, whereas the latter has a clear preference for fast stimuli. It has been shown that dopaminergic neurons in the posterior tuberculum (PT) respond reliably to tactile stimulation. Their velocity tuning is very similar to the one we determined for the anterior lateral line cells. Furthermore, when all hair cells of the lateral line are chemically ablated, some of the PT’s dopaminergic neurons lose their velocity tuning (Reinig et al., 2017). Both results, taken together, indicate that the anterior lateral line system is excited by tactile stimulation and sends those signals to diencephalic dopaminergic clusters via pathways that have yet to be identified.

It was surprisingly difficult to obtain calcium imaging recordings of the anterior lateral line neurons during tactile stimulation, especially when we attempted to stimulate the animal with water flow. Although posterior lateral line afferent neurons have been recorded quite successfully in the past using different sets of stimulation and recording methods (Liao and Haehnel, 2012; Haehnel-Taguchi et al., 2014; Levi et al., 2015; Odstrcil et al., 2022), there are only a few reports on the activity of the anterior lateral line in fish. For example, there are electrophysiological recordings of the anterior lateral line in long-fin eel *Anguilla dieffenbachia* (Voigt et al., 2000), the oyster toadfish *Opsanus tau* (Palmer et al., 2005), and the Antarctic fish *Pagothenia borchgrevinki* (Montgomery and Macdonald, 1987). We present, here, the first physiological recordings of the zebrafish’s anterior lateral line neurons in response to a water flow stimulus. Both recorded neurons show a clear direction sensitivity, which reflects the selectivity of hair cells and posterior lateral line neurons (López-Schier et al., 2004; López-Schier and Hudspeth, 2006; Nagiel et al., 2008; Faucherre et al., 2009).

To improve the construction of robots, engineers have been inspired by nature and its solutions to specific problems. In building underwater robots, it seems to be a promising idea to seek guidance from the mechanisms that are used by fish and other underwater dwelling animals. The biological systems that we can observe today have proven to be useful in a variety of circumstances and were shaped by evolution over thousands of years. One possibility of incorporating such principles is, for example, building artificial water flow sensors and processing algorithms that are based on the lateral line system and its mechanical structures and neuronal computations (Tao and Yu, 2012; Liu et al., 2016; Haehnel-Taguchi et al., 2018a). Compared to the posterior lateral line that is organized linearly along the body axis, we hypothesize that the anterior lateral is much more capable of encoding three-dimensional flow fields, helping the animal to get more information about the subtle changes of water flow around its head. The anterior lateral line afferent neurons that possess a fine-tuned responsiveness to a certain range of stimulus velocities could be used as a blueprint for optimizing the sensory range of artificial flow sensors. Since robots tend to get smaller in size, knowledge from miniature animals like the zebrafish larva could help in optimizing the architecture, materials, and computational processing steps involved in designing small-scale robots (Haehnel-Taguchi et al., 2018a). Zebrafish larvae are not larger than 5 mm, and evolution has optimized their sensory apparatus for this specific small body size. The physical properties of fluids are scale-dependent, and thus, it is sensible to mimic the design mechanisms of such a small animal in constructing small robots.

The lateral line is, however, not the only system participating in mechanosensation. As we could show, the trigeminal system, with its fine nerve endings innervating the skin, delivers useful information for the animal’s nervous system as well. The lateral line and trigeminal system, together, cover a certain range of stimulus properties. These properties are encoded in the system activity that can be further analyzed, combined, and integrated in later stages. Therefore, it might be fruitful to combine several different kinds of sensors (modalities) to get a more detailed and finer “image” of the surrounding environment and to improve the important task of discrimination between self-induced water motion and external stimulation. This could be even extended to a multi-modal processing system that combines several different sensor systems like pressure, flow, sound, and vision to optimally navigate through water. Such a multi-modal approach increases the overall signal-to-noise ratio by integrating information from different sensory channels that are all optimized to encode a specific range of physical properties. Therefore, the principle of having several independent sensory systems that cover a wide range of environmental stimuli that can be compared and integrated in later processing stages can be adapted in designing more complex and sophisticated sensor banks for guiding the control of underwater robots. In the future, in particular, the organized anatomical and functional structure of the anterior lateral line neuromasts along the head and around the eyes (Chou et al., 2017) could be intriguing to better understand how to optimize the location of sensors and their internal mode of operation (Yanagitsuru et al., 2018).

## Data Availability

The raw data supporting the conclusion of this article will be made available by the authors, without undue reservation.
